# Excessive anterior tibial translation in the contralateral uninjured limb is significantly associated with ramp lesion in anterior cruciate ligament injury

**DOI:** 10.1186/s40634-021-00372-9

**Published:** 2021-07-23

**Authors:** Kazuki Asai, Junsuke Nakase, Rikuto Yoshimizu, Mitsuhiro Kimura, Hiroyuki Tsuchiya

**Affiliations:** Department of Orthopaedic Surgery, Graduate School of Medical Science, Kanazawa University, 13-1 Takara-machi, Kanazawa, 920-8641 Japan

**Keywords:** Anterior cruciate ligament injury, Ramp lesion, Meniscus injury, Excessive anterior tibial translation, Muscle strength

## Abstract

**Purpose:**

This study aimed to evaluate the excessive anterior tibial translation (ATT) and muscle strength of patients with ramp lesions. We hypothesised that the higher ATT, lower hamstring-to-quadriceps (HQ) ratio, and higher flexion peak torque influenced by semimembranosus would be associated with ramp lesions.

**Methods:**

One hundred and twenty-one patients who underwent anterior cruciate ligament (ACL) reconstruction were retrospectively evaluated. Clinical evaluation included ATT of the contralateral uninjured limb measured using a KT-1000 arthrometer, the knee flexor and extensor muscle strength of the contralateral uninjured limb at 60°/s and 180°/s of an angular velocity measured using an isokinetic dynamometer, and HQ ratio at 60°/s and 180°/s during the preoperative state. Binary stepwise logistic regression analysis was performed to evaluate the risk factors of ramp lesions.

**Results:**

Ramp lesions were found in 27 cases of ACL injuries (27/121, 22.3%). Male sex (odds ratio [OR], 2.913; 95% confidence interval [CI], 1.090–7.787; P = 0.033), longer time between injury to surgery (OR, 2.225; 95% CI, 1.074–4.608; P = 0.031), and higher ATT in the contralateral uninjured limb (OR, 1.502; 95% CI, 1.046–2.159; P = 0.028) were indicated as the independent risk factors of the presence of ramp lesion associated with an ACL injury.

**Conclusions:**

Male sex, longer period from injury to surgery, and higher ATT in the contralateral uninjured limb were significantly associated with ramp lesion. These findings are advantageous for identifying patients with a greater risk of developing a ramp lesion with an ACL injury in the clinical setting.

**Level of evidence:**

Level IV

## Background

Ramp lesion is a specific type of meniscus lesion such as a meniscocapsular tear of the posterior horn of the medial meniscus (PHMM) associated with anterior cruciate ligament (ACL) injury, which is called a “hidden lesion” [18]. Ramp lesions accounted for 9%–24% of all ACL injuries [2, 10, 19]. Some studies have reported that longer time from injury to surgery, young age, and contact injury mechanism were risk factors for ramp lesions [1, 10, 19]. In addition, George et al. reported that the presence of bone marrow oedema of the posteromedial tibia or a lateral meniscal tear was associated with the presence of ramp lesions [1]. However, the mechanism and risk factors of ramp lesions remain unclear.

While the precise mechanisms through which these lesions manifest are not entirely understood, some studies have hypothesised that excessive anterior tibial translation (ATT) and semimembranosus contraction in response to ACL injury possibly cause a ramp lesion [4, 7, 17, 21]. Biomechanical studies have shown that ATT increases following ACL injury, allowing the PHMM to be trapped between the femoral and tibial condyles, acting as a wedge against the tibia, thereby significantly increasing forces in the PHMM [9, 11, 13]. The quadriceps and hamstrings apply antagonist forces to the tibia and play an important role in knee stabilisation [16]. Therefore, imbalance of the quadriceps and hamstring muscles strength potentially causes excessive ATT [6].

To the best of our knowledge, only a few studies have focused on knee laxity and muscle strength in patients with ramp lesions. We aimed to evaluate the characteristics of patients with ramp lesions with a special focus on excessive ATT and hamstring and quadriceps strength. Since it was difficult to assess muscle strength precisely in ACL-injured knees during the preoperative state due to pain and fear, we assessed muscle strength of the contralateral uninjured limb in this study. We hypothesised that the higher ATT, lower hamstring-to-quadriceps (HQ) ratio, and higher flexion peak torque influenced by semimembranosus would be found in patients with ramp lesions associated with an ACL injury.

## Methods

### Study design and patients

One hundred sixty-one patients who underwent ACL reconstruction at our hospital between January 2017 and December 2020 were retrospectively evaluated. ACL injury was diagnosed using magnetic resonance imaging (MRI) and clinical assessments such as the Lachman test and pivot shift test. In all patients, the quadriceps and hamstrings muscle strengths were assessed on the day before surgery. ATT of the uninjured contralateral knee was also assessed as it was difficult to infer the true ATT of the ACL-injured knee during the preinjury state. A previous study found < 3% difference in peak torque values between dominant and nondominant limbs, which is the same as the accuracy of the force measuring system of isokinetic torques of 3% [5]. Therefore, we considered that it might be helpful to measure the muscle strength of the contralateral uninjured limb to estimate the strength of the injured limb. The exclusion criteria were ACL re-injury, multi-ligament injuries requiring other ligament surgeries, and history of contralateral ACL injury or other knee surgery. The cases in which muscle strength assessment could not be performed before surgery were also excluded. After excluding 40 patients, 121 cases were finally analysed (age, 23.5 ± 11.6 years; body mass index [BMI], 23.0 ± 3.3 kg/m^2^; and time from injury to surgery, 80 ± 93 days). The clinical assessments were compared between the ramp and non-ramp lesion groups. This study was approved by the ethics committee of our hospital (2745–2), and informed consent was obtained from each patient.

### Surgical technique

**Fig. 1:**
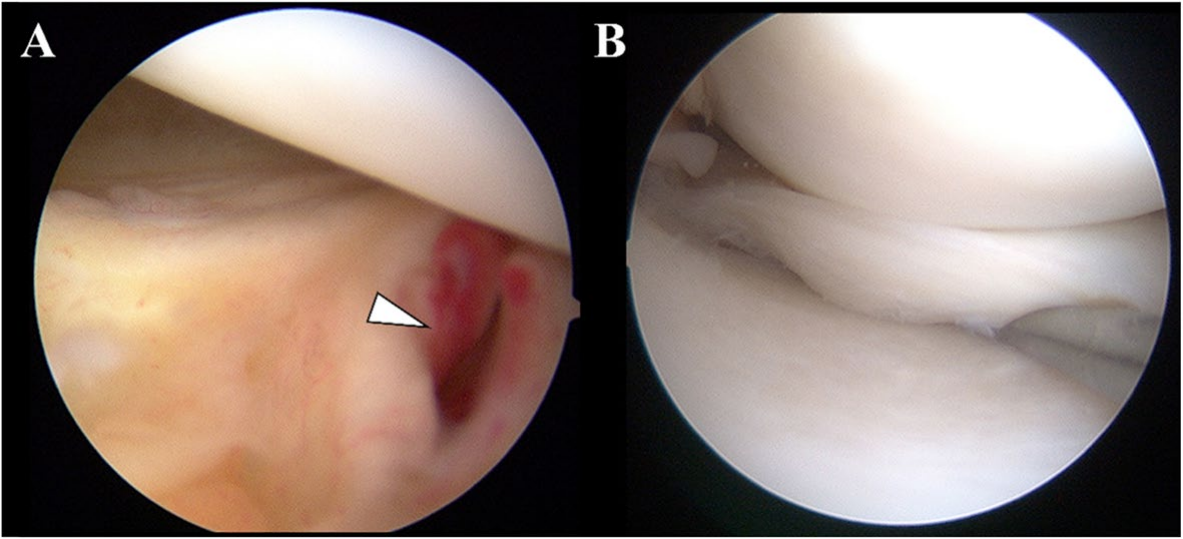
Systematic arthroscopic evaluation of ramp lesions. **A** The meniscocapsular junction is directly assessed by intercondylar viewing. **B** Second, the medial meniscus is evaluated through the anterolateral portal with probing. Arrowhead marks meniscocapsular tear of the posterior horn of the medial meniscus

All surgeries were performed by a single senior surgeon. The medial and lateral meniscal lesions were evaluated before ACL reconstruction using 30° arthroscopes via an anterolateral portal with probing. The medial meniscus was assessed at 90° of flexion, and the lateral meniscus (LM) was assessed in the figure-of-4 position. After systematic arthroscopic evaluation for meniscal lesions, the presence of ramp lesions was assessed [20]. First, the meniscocapsular junction was directly assessed by intercondylar viewing with 30° arthroscope; in some cases, probing was performed through the posteromedial portal. The arthroscope was passed through the anterolateral portal and inserted between the posterior cruciate ligament and the medial wall of the intercondylar notch into the posteromedial compartment with the knee in 90° flexion and slightly externally rotated (Fig. 1A). Inspection through the posteromedial portal may detect more ramp lesions [18], but this requires more time and is significantly invasive for a screening procedure. Therefore, this technique was used in only suspected cases as needed. Second, the medial meniscus was evaluated through the anterolateral portal with probing (Fig. 1B).

### Clinical assessment

Clinical evaluation included ATT of the contralateral uninjured limb, the knee flexor and extensor muscle strength at 60°/s and 180°/s of angular velocity, and the HQ ratio at 60°/s and 180°/s during the preoperative state. ATT was measured by a KT-1000 arthrometer (MEDmetric Corp., San Diego, CA, USA) with manual max by the orthopaedic surgeon who was blinded to any other information, such as MRI findings. A physiotherapist who was also blinded to any other findings used an isokinetic dynamometer (BIODEX System 4; BIODEX Company, Shirley, NY, USA) to assess flexion and extension peak torque at 60°/s and 180°/s. After warming up using an ergometer for 5 min and practicing two sets of flexion and extension exercises thrice, the patients participated in the measurement test. The patients were allowed sufficient rest time between the sets. The measurement range was 0°–100° for the knee joint, and the lower leg was gravity-corrected. The average knee flexion and extension peak torque were calculated by three flexion and extension exercises at the production set. The HQ ratio was defined as hamstring muscle strength (flexion peak torque)/quadriceps muscle strength (extension peak torque).

### Statistical analysis

Continuous variables were presented as mean ± standard deviation. According to the results of Shapiro–Wilk test used to test the normality of their distribution, age, BMI, ATT of the uninjured contralateral limb, and the flexion peak torque at 180°/s were compared using Mann–Whitney U test between the ramp and non-ramp lesion groups. Chi-squared test was used to compare sex, time from injury to ACL reconstruction (< 3, 3–6, or > 6 months), LM tear, and mechanism of injury (contact or non-contact) between the groups. The flexion peak torque at 60°/s, extension peak torque at 60°/s and 180°/s, and the HQ ratio at 60°/s and 180°/s were compared using independent *t* test. Binary stepwise logistic regression analysis was performed to evaluate the risk factors of the presence of ramp lesions. The predictive factors were as follows: age at surgery, sex, BMI, time from injury to ACL reconstruction (< 3, 3–6, or > 6 months), LM tear, mechanism of injury (contact or non-contact), ATT in the uninjured contralateral limb, the knee flexion and extension peak torque at 60°/s and 180°/s, and the HQ ratio at 60°/s and 180°/s during the preoperative period. The significance level was set at p < 0.05, and the 95% confidence interval (CI) was calculated. All statistical analyses were performed using IBM SPSS Statistics for Windows, ver. 24 software (IBM Corp., Armonk, NY, USA).

Post-hoc power analysis was performed with a chi-squared goodness-of-fit test with an α error of 0.05 and a β error of 0.2 to detect the differences in the incidence of significant risk factors between the groups. Based on these calculations, the power of significant factors in this study was > 0.9. To detect differences in mean values of significant risk factors between the groups, post-hoc power analysis was also performed with an α error of 0.05 and a β error of 0.2. The power of the mean values of significant risk factors was > 0.8. These post-hoc power analyses were performed using G* power 3.1 software (Heinrich-Heine University Dusseldorf, Dusseldorf, Germany).

## Results

**Fig. 2:**
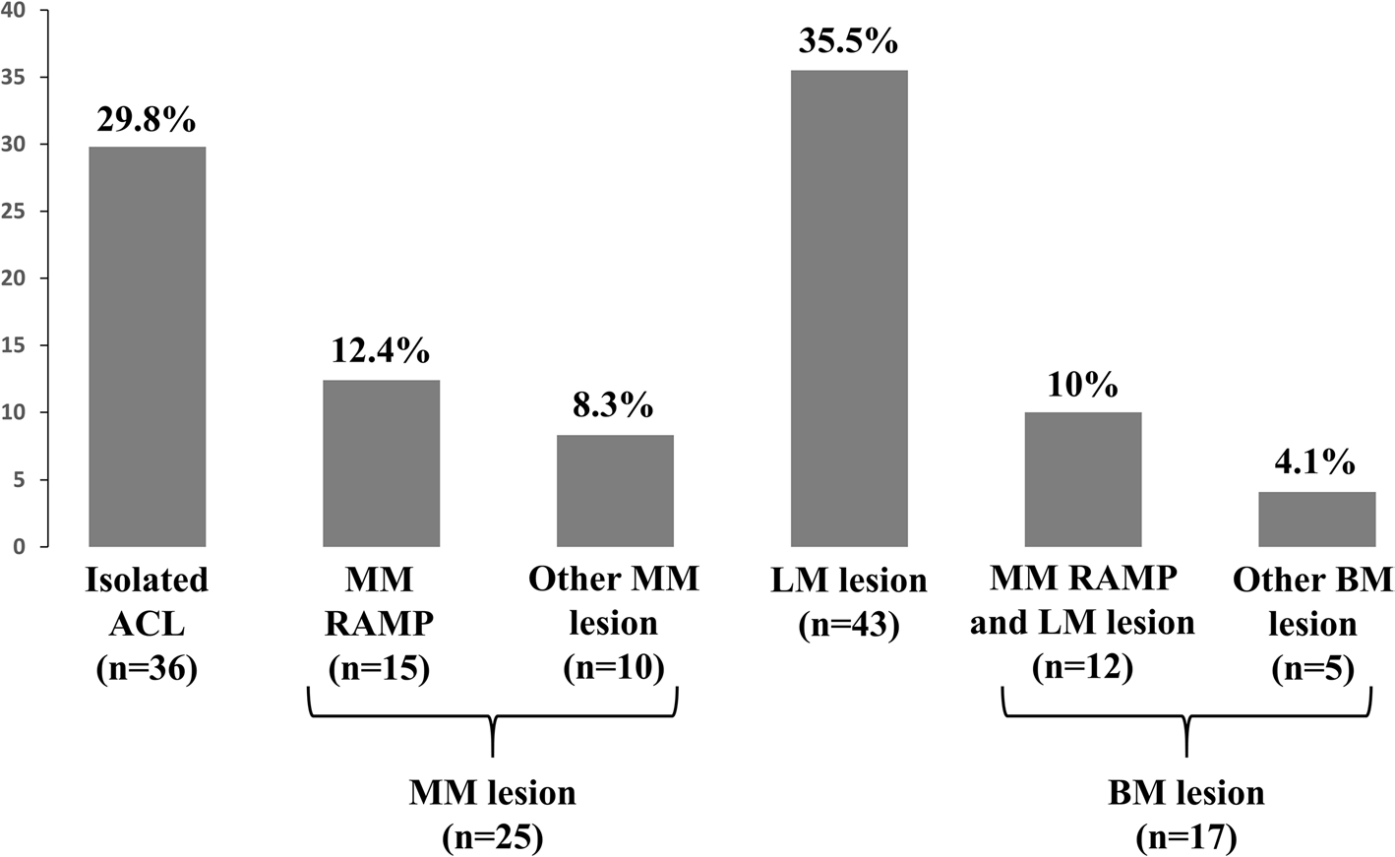
Prevalence of concomitant meniscal lesion with an ACL injury. Meniscus injuries and specific types of lesions, including ramp lesions, are presented. MM, medial meniscus; LM, lateral meniscus; BM, bimeniscal; ACL, anterior cruciate ligament

In this study, 70.2% (85/121) of ACL injury cases had a meniscal tear (Fig. 2). Ramp lesions were found in 27 cases of ACL injury (27/121, 22.3%). Among the cases of ramp lesion, 44.4% (12/27 cases) had a concomitant LM tear.

### Demographics of patients with a ramp lesion

The characteristics of patients with and without a ramp lesion are presented in Table 1. The ramp lesion group showed significantly higher predilection in men, longer time from injury to surgery, and higher ATT in the contralateral uninjured limb than the non-ramp lesion group (P = 0.044, 0.001, and 0.034, respectively).

**Table 1:** Patient demographics

	Ramp lesion (27 cases)	Non-ramp lesion (94 cases)	P-value
Age (y)	21.9 ± 8.0	24.0 ± 12.4	n.s
Sex (Male:Female)	18:9	43:51	**0.044**
BMI (kg/m^2^)	22.4 ± 2.1	23.0 ± 3.4	n.s
Time from injury to surgery (months)	0–3: 14	0–3: 79	**0.001**
	3–6: 11	3–6: 10	
	> 6: 2	> 6: 5	
Lateral meniscus tear	12 (44.4%)	47 (50.0%)	n.s
Mechanism of injury (Contact:Non-contact)	5:22	30:64	n.s
Anterior tibial translation (mm)	5.4 ± 1.6	4.7 ± 1.1	**0.034**
Flexion peak torque (60°/s)	59.5 ± 21.8	59.6 ± 21.2	n.s
Extension peak torque (60°/s)	140.3 ± 40.1	133.8 ± 42.5	n.s
HQ ratio (60°/s)	0.42 ± 0.09	0.45 ± 0.09	n.s
Flexion peak torque (180°/s)	52.0 ± 20.4	51.0 ± 18.8	n.s
Extension peak torque (60°/s)	94.5 ± 27.7	90.9 ± 30.5	n.s
HQ ratio (180°/S)	0.54 ± 0.10	0.56 ± 0.10	n.s

*BMI* body mass index, *HQ* hamstrings strength-to-quadriceps strength, *n.s*., not significant

### Risk factors of ramp lesions

On logistic regression analysis, male sex (odds ratio [OR], 2.913; 95% CI, 1.090–7.787; P = 0.033), longer time from injury to surgery (OR, 2.225; 95% CI, 1.074–4.608; P = 0.031), and higher ATT in the contralateral uninjured limb (OR, 1.502; 95% CI, 1.046–2.159; P = 0.028) were indicated as independent risk factors for the presence of ramp lesions associated with ACL injury (Table 2).

**Table 2:** Results of logistic regression analysis of ramp lesions

	Odds ratio	β	SE	95% CI	P-value
Sex (male)	2.913	1.069	0.502	1.090–7.787	**0.033**
Time from injury to surgery	2.225	0.800	0.371	1.074–4.608	**0.031**
Anterior tibial translation	1.502	0.407	0.185	1.046–2.159	**0.028**
HQ ratio	0.006	-5.190	2.688	0.000–1.081	n.s

Likelihood ratio test, P = 0.001

Nagelkerke R^2^ = 0.225

*SE* standard error, *CI* confidence interval, *HQ* hamstrings strength-to-quadriceps strength, *n.s*., not significant

## Discussion

On logistic regression analysis, male sex, long time from injury to surgery, and higher ATT in the contralateral uninjured limb were significantly associated with ramp lesions. Although some of our hypotheses were confirmed, others about muscle strength were not.

This study also found excessive ATT in the contralateral uninjured limb as an indicator of knee condition during the pre-ACL injury state and as a risk factor of ramp lesion. Our findings were supported by those of previous studies that advocated a similar mechanism for developing a ramp lesion: excessive ATT and semimembranosus contraction during ACL injury may lead to excessive loading of the PHMM, meniscotibial ligament, and meniscocapsular junction, resulting in a ramp lesion [4, 7, 17]. Saygi et al. found that semimembranosus reflexively contracts in response to PHMM translation during ATT and is responsible for posterior shifting of the PHMM [7, 15]. Therefore, we considered that the patients who presented with excessive ATT in the contralateral uninjured limb carry a risk for ramp lesions even if the preoperative side-to-side laxity is not high. The incidence of ramp lesions in our study was similar to that in a previous large cohort study by Sonnery et al., who reported ramp lesions in 23.9% (769/3214 cases) of ACL injury cases [19]. They revealed that male sex and longer chronic period between injury to surgery were risk factors of ramp lesions [19]. However, a previous study also found that young age, contact injury, and concomitant LM injury were risk factors of ramp lesions, whereas these factors were not significant in the present study [1, 19].

We also explored the semimembranosus contraction as one of the causes of ramp lesions. Recently, many studies have investigated the association between the semimembranosus muscle and ramp lesions [3, 4, 21]. Based on histological findings, DePhillipo et al. reported that the branch from the anterior arm of the semimembranosus muscle tendon is attached to the posterior inferior margin of the medial meniscus [4]. Vieira et al. performed an arthroscopic assessment and found that applying load to the semimembranosus tendon leads to posterior translation of the PHMM and stretching of the meniscocapsular region [21]. Cavaignac et al. noted that the capsular arm of the semimembranosus tendon protruded over the joint capsule, and adipose tissue was located in between the capsular branch of the semimembranosus and the medial meniscus, including the common attachment of the meniscocapsular and meniscotibial ligaments [3]. However, contrary to our hypothesis, there was no significant difference in the peak torque of knee flexion, which may be influenced by the semimembranosus muscle. The capsular branch of the semimembranosus was subjected to the milder mechanical stress than the direct arm of the semimembranosus inserted into the tibia because of their secondary mechanical role [3]. Therefore, our evaluation methods of merely assessing the hamstrings muscle strength might have been inadequate to detect the difference in activity of the capsular branch of the semimembranosus between the knees with and without ramp lesions.

The findings of the present study should be interpreted with consideration of few limitations. First, this study had possible selection bias due to the exclusion of patients in whom muscle strength was not assessed. Second, there was a relatively small number of cases in this study. Third, we evaluated the muscle strength of contralateral uninjured limbs as an indicator of the knee condition during the pre-ACL injury state. Previous studies found less than 3% differences in the peak torque value and 1% differences in the HQ ratio between dominant and nondominant legs [5, 12, 14]. Lee et al. demonstrated a 50% reduction of thigh muscle strength in a group of patients with chronic ACL injury. However, they still observed HQ ratios comparable to those of a healthy control group [8]. Therefore, we considered that evaluating the HQ ratio of the contralateral uninjured limb was rather reasonable for assessing the muscle strength of knee extension and flexion before ACL reconstruction. Fourth, this study did not consider the influence of time from injury to surgery. A previous study reported decreased muscle strength in chronic ACL-injured knees [8]. Despite these limitations, the results of this study suggested that the patients with a predisposition to excessive ATT due to knee laxity or a lower HQ ratio carry the risk of developing a ramp lesion with an ACL injury. Moreover, a long time from injury to surgery was a risk factor of ramp lesions. These findings might help surgeons counsel patients in the setting of ACL tears with concomitant ramp lesions.

## Conclusions

Male sex, a longer period from injury to surgery, and higher ATT of the contralateral uninjured limb were significantly associated with ramp lesions. These findings are advantageous for identifying patients with a greater risk of developing a ramp lesion with an ACL injury in the clinical setting.

## Data Availability

The datasets used and analysed during the current study are available from the corresponding author on reasonable request.

## Abbreviations

ACL: Anterior cruciate ligament
ATT: Anterior tibial translation
BMI: Body mass index
CI: Confidence interval
HQ: Hamstring-to-quadriceps
LM: Lateral meniscus
MRI: Magnetic resonance imaging
OR: Odds ratio
PHMM: Posterior horn of the medial meniscus
